# Patterns of Rising HIV Positivity in Northern Madagascar: Evidence of an Urgent Public Health Concern

**DOI:** 10.3390/tropicalmed9010019

**Published:** 2024-01-11

**Authors:** Kyle E. Robinson, Jackson K. Long, Mamantsara Fardine, Adriantiana M. Stephano, Andrew Walsh, Eric P. Grewal

**Affiliations:** 1 Mada Clinics, Maventibao, Madagascar; 2 Department of Anthropology, University of Western Ontario, London, ON N6A 3K7, Canada; 3 Department of Pathology, Massachusetts General Hospital, Boston, MA 02114, USA

**Keywords:** HIV, prevalence, Madagascar, point of care, epidemic

## Abstract

Despite over two decades of progress against HIV/AIDS in adjacent sub-Saharan Africa, HIV rates and deaths due to AIDS are exponentially rising in Madagascar. Furthermore, a growing body of evidence suggests that, due to a scarcity of general-population screening data, even the startling increase demonstrated by official models vastly underestimates the true population prevalence of HIV. We aimed to implement a real-world HIV screening and treatment protocol to serve a general population stemming from across northern Madagascar. In collaboration with the Malagasy Ministry of Health, we provided point-of-care HIV screening and confirmatory testing for over 1000 participants from 73 towns, villages, and cities. We recorded an overall HIV prevalence of 2.94%. Notably, we observed a 13.1% HIV prevalence rate among urban populations and showed that proximity to a major route of travel was significantly associated with HIV risk. We also observed a link between HIV risk and various occupations, including those associated with increased mobility (such as mining). Importantly, all HIV-positive individuals were initiated on antiretroviral therapy in concordance with local health authorities. To our knowledge, this study marks the largest primary test data-based HIV study to date among Madagascar’s general population, showing a greatly higher HIV prevalence (2.9%) than previously reported modeling-based figures (0.4%). Our rates aligned with the pattern of higher prevalence demonstrated in smaller general-population screening studies occurring more commonly prior to political strife in the mid-2000s. These findings demonstrate evidence of a growing HIV epidemic in northern Madagascar and underscore the need for future investment into more comprehensive HIV screening and control initiatives in Madagascar.

## 1. Introduction

Since the year 2000, data reported by the Joint United Nations Program on HIV/AIDS (UNAIDS) have demonstrated a rapid and exponential rise in HIV cases and AIDS-related deaths in Madagascar, an island nation with a population that exceeds 28 million [1]. Between 2000 and 2022, the number of people living with HIV in Madagascar has increased by 3400% (from 2000 to 70,000), the yearly incidence of new HIV cases has increased by 650% (from 0.04 to 0.3 per 1000), and annual deaths due to AIDS have risen by approximately 3100% (from <100 to 3200). These rates appear to be accelerating despite successful programs to combat HIV/AIDS in much of the rest of the African continent over the past two decades [2].

A major impediment to HIV control in Madagascar was a 2008 coup d’état that led to the suspension of many national health and foreign aid programs. This coup was followed by an interim administration with high levels of instability, lacking sufficient resources for HIV research, prevention, and treatment [3]. As the Malagasy Ministry of Health recently began to reorganize and reinitiate public health programs, the COVID-19 pandemic again suspended many initiatives, and SARS-CoV-2 became the central public health focus since its emergence [4].

Though the exponential increase in HIV rates is startling, HIV research in Madagascar is extremely limited, and available rates are largely based on what little data could be collected prior to the turmoil of recent decades. A 2020 meta-analysis on the current state of HIV surveillance in Madagascar concluded that it is not possible to accurately determine the rate of HIV in the country based on the scarcity of data, and that the outputs of disease modeling should be interpreted with a high level of caution due to the lack of accurate epidemiologic data to use as inputs [5]. This conclusion supports a growing consensus that official rates (based on Spectrum modeling) could deeply underestimate the extent of HIV infection in Madagascar, even in light of the rapid rise in cases that they already demonstrate. Another weakness of available studies is that most HIV data are derived from select “key” populations in urban centers, despite Madagascar having a majority rural population [5,6]. Furthermore, health policies in Madagascar require HIV tests and antiretroviral medications to be supplied through the Ministry of Health [7]. Thus, clinics outside of well-connected urban centers or independent of national programs are often unable to test patients for HIV or provide treatment if patients test positive [8]. This likely substantially hindered the implementation of HIV treatment and research throughout the coup and subsequent interim period.

Adding to these concerns, a recent audit performed by the Global Fund in 2021 identified multiple critical gaps in the country’s HIV/AIDS control strategies, including at grant-supported antiretroviral therapy clinics [9]. Shockingly, no HIV tests were available at any of the antiretroviral treatment facilities visited by auditors, a major hindrance to confirming the HIV status of any patients. The auditors also identified multiple inconsistencies in data reporting and a substantial lack of clarity regarding how HIV data are initially obtained and relayed to arrive at existing prevalence estimates. Since the publication of this report, the Malagasy Ministry of Health has recently begun expanding resources for HIV testing and antiretroviral treatment, but challenges remain in ensuring these provisions are accessible to a large proportion of citizens.

One observation that may contribute to a high burden of HIV in Madagascar includes anthropological studies that report high rates of transactional sex across multiple regions of Madagascar, with approximately one-third of young females living in urban regions and two-thirds of those living in rural regions reportedly engaging in relations for monetary compensation [10]. Relatedly, Madagascar carries one of the highest per capita burdens of gonorrhea, chlamydia, and syphilis infection in the world [6,10], suggesting that HIV rates should track similarly high. Yet, Spectrum model-derived figures thus far utilized by the Ministry of Health had placed the HIV prevalence rate uniquely low, at 0.4% [5,11]. In addition to high sexually transmitted infection (STI) rates, another factor raising suspicion of elevated HIV rates is the extensive proportion of the populace involved in mining (>10%), where travel in pursuit of gem or precious metal deposits serves as a means of geographically disseminating HIV [12,13].

The Institut Pasteur provided one major rural HIV prevalence estimate during a cross-sectional study conducted in 2002 across Madagascar’s northwest coast, which reported an HIV rate of approximately 1% [6]. However, no large general-population screening studies have been published in the intervening two decades. With the absence of major coordinated HIV/AIDS-related interventions through this period, there is considerable concern that rates could have vastly increased beyond those observed by the Institut Pasteur, and that such an increase is not encompassed by current data.

Mada Clinics is a Malagasy government-certified healthcare center that provides free care for a large portion of rural northern Madagascar, receiving patients from nearly 75 towns, villages, and cities. Over the past seven years, the center noted an increasing burden of patients with fungal infections, recurrent fevers, and chronic weight loss, suggesting a clinical need to initiate HIV screening.

After completing a four-year process of certification with the Malagasy Ministry of Health, we (Mada Clinics) initiated a screening and treatment protocol for HIV into all primary care visits in order to assist the Ministry of Health in collecting much needed population screening data. We posited that this current study might yield valuable, general-population, in-person testing data for use by the Ministry of Health, as existing efforts have focused on limited “key” populations. Furthermore, we aimed to assess how HIV rates may have changed in rural Madagascar over the past twenty years.

Given that the Mada Clinics patient population includes adolescents and adults presenting for low-acuity care, we posited that this group may serve as a more representative population than patients seen in private clinics or secondary/tertiary care centers. Building upon the work of the Institut Pasteur, whose notable study surveyed 16 rural villages, this investigation included over one thousand patients from 73 towns, villages, and cities across northern Madagascar. To our knowledge, this is the largest HIV prevalence study conducted among the general population of Madagascar to date.

## 2. Materials and Methods

### 2.1. Patient Enrollment

This study was conducted within a two-month interval between October 2022 and March 2023, during which, all patients presenting to the clinic for routine care were asked whether they would like HIV screening. Patients were enrolled into the study daily until over 1000 total patients were accrued. All participants consented verbally prior to study enrollment, including consent from parents for minors. Prior to testing, all patients were counselled about basic HIV biology, its mechanism of transmission, clinical consequences of infection, and treatment options. Patients were also queried to screen out individuals with known positive HIV status or those who had received prior antiretroviral therapy. Biographic and demographic data were collected from each subject and tabulated in an anonymized fashion. Patients were not compensated for participation in this study, though all testing, care, and treatment were provided free of charge. This study was conducted in accordance with the Declaration of Helsinki and was authorized by the Malagasy Ministry of Health in Antsiranana, including approval by their Ethics Committee. To ensure proper cultural sensitivity and acceptance by local communities, additional authorization was sought from the Ethics Committee for Public Health Research in northern Madagascar, a multidisciplinary committee including local leaders representing the farmers, miners, women, and youths in rural northern Madagascar. The committee also includes anthropologists and public health leaders. With their support, a high degree of trust was engendered amongst the 73 sampled locations, and a greater than 99% rate of consent to testing was encountered.

### 2.2. Testing Procedure

Patients who consented to study enrollment were tested using a two-tiered testing algorithm employing oral fluid or fingerstick blood samples. Screening was performed by using one of four commercially available, qualitative point-of-care assays for the rapid detection of HIV-1/2 antibodies (Table 1). Patients who screened positive for HIV antibodies underwent confirmatory testing using the SURE CHECK^®^ HIV assay (Chembio Diagnostics, Medford, NY, USA), a U.S. FDA-cleared immunochromatographic device that provides superior sensitivity and specificity for HIV antibody detection from fingerstick blood [14]. Additional immunoassay-based confirmatory testing was often conducted by the Ministry of Health, leading to positive patients receiving an average of three tests prior to the initiation of antiretroviral therapy. All samples were obtained and tested by a trained medical staff member. The results were tabulated as positive or negative based on manufacturer instructions for each assay. Test kits were provided for use by the Malagasy Ministry of Health, with additional financial support from Mada Clinics.

### 2.3. Treatment and Follow-Up

In accordance with local health directives, patients who were confirmed to be HIV-positive were registered with the local Ministry of Health in Anstiranana. Following guidance from a ministry-authorized infectious disease physician, HIV-positive patients were prescribed one of two available combination antiretroviral regimens (Table 2). All medications were furnished by the Malagasy Ministry of Health and were dispensed by credentialed clinic staff. HIV-positive patients were scheduled for regular monthly follow-up at Mada Clinics. Patients were also provided the option of referral to a subspecialty care center in Antsiranana, where additional laboratory monitoring capabilities were available.

### 2.4. Statistical Considerations

Clinical data were tabulated using paper records and transferred to a secure digital database upon study completion. Statistical analysis was performed using Graphpad Prism (Version 9; GraphPad Software, San Diego, CA, USA). For the comparison of independent groups, a two-tailed Fisher’s exact test was employed. Significance for all comparisons was defined as *p* < 0.05.

## 3. Results

### 3.1. Patient Demographics and HIV Testing Results

**Figure 1:**
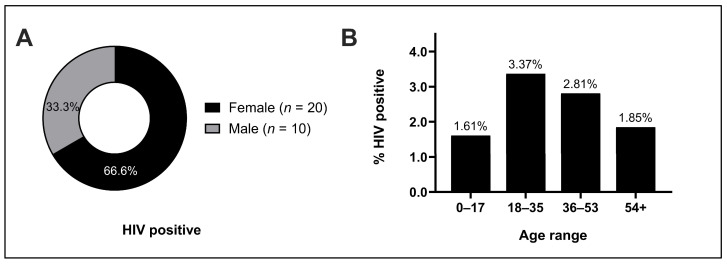
Demographics of HIV-positive individuals. (**A**) Total number of HIV-positive individuals enumerated by sex, and (**B**) rate of HIV positivity in study population by age group.

During the study period, 1026 patients presented for care, 1019 of whom consented to undergo HIV screening and study enrollment. No patients were known to have previously tested positive or received treatment for HIV. In total, 30 patients tested positive on all screening and confirmatory tests, representing an overall HIV prevalence rate of 2.94% (Table 3). By gender, 66.7% (20/30) of positive cases occurred in females, representing a 3.47% positivity rate in female patients and a 2.26% positivity rate in male patients (Figure 1A). HIV positivity was greatest in patients in the 20s to 30s age range, with a mean age of 35.2 years (Figure 1B). Of note, only one individual under the age of 18 tested positive for HIV. There were no discrepancies between screening and confirmatory test results.

### 3.2. Geographic and Sociographic Patterns

**Figure 2:**
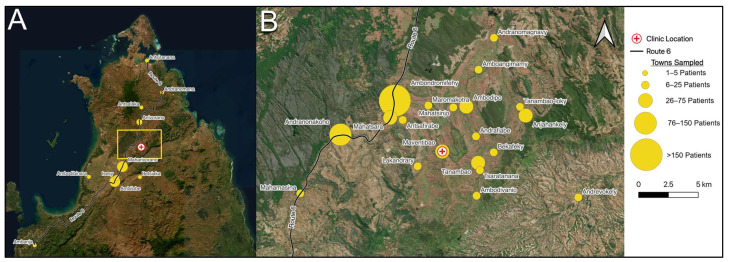
Geographic distribution of HIV prevalence. (**A**) Large-scale and (**B**) detailed view of villages, towns, and cities from which HIV-positive individuals were identified. Photos courtesy of Google Earth.

**Figure 3:**
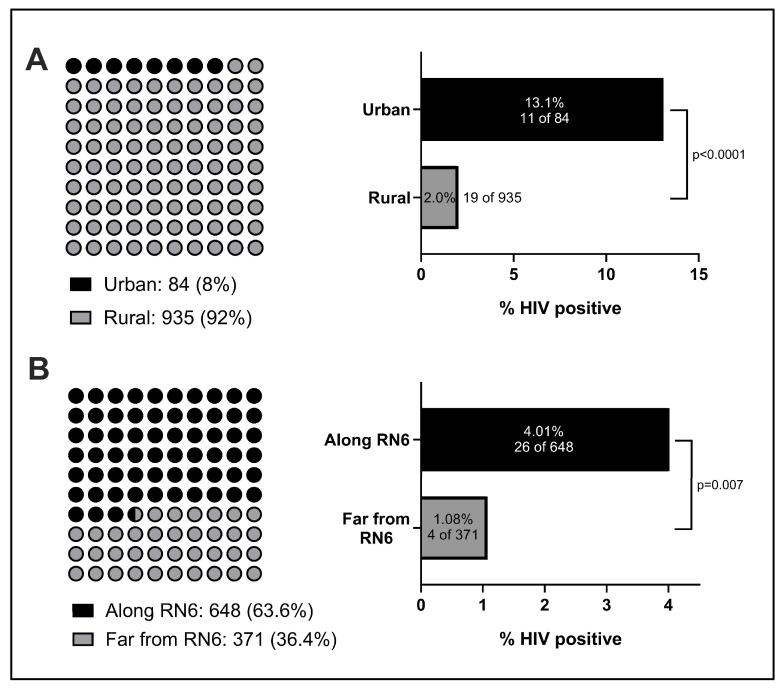
**Geographic distribution of HIV prevalence.** (**A**) Total number of study participants from urban or suburban locales (black) versus rural villages (gray) and proportion of HIV-positive individuals originating from each geography. (**B**) Number of individuals living close to (black) or far from RN6 (gray) and percent of HIV-positive individuals originating from each locale. Each dot represents roughly 10 study participants.

Patients presented from 73 different towns, villages, or cities (Figure 2). While the majority of patients lived in rural villages, 8.2% (84) of patients presented from cities or suburbs (“urban”) (Figure 3A). Among this urban population, the HIV prevalence was significantly greater (13.1%) than among the rural population, where 2.03% were found to be HIV-positive. We hypothesized that increased mobility and access to major routes of transportation could be associated with HIV infection. One major transportation route is Route Nationale 6 (RN6), the primary highway in northern Madagascar that spans 706 km from Antsiranana to Ambondromamy. The majority of patients were found to live along RN6, and among this population, the HIV prevalence was significantly greater compared to those living away from RN6 (Figure 3B). Strikingly, all (10/10) HIV-positive men lived along RN6, with an overall HIV rate of 3.40% in this segment. In contrast, no males living away from RN6 (0/148) tested positive for HIV, despite this demographic constituting a major subpopulation of the study.

**Figure 4:**
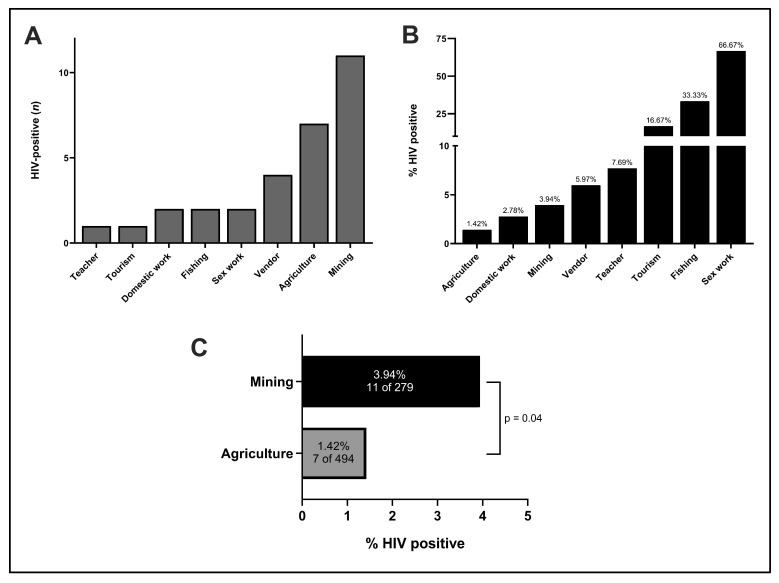
HIV status by occupation. (**A**) Occupations held by individuals who tested positive for HIV, (**B**) percentages of HIV-positive individuals by occupation, and (**C**) HIV-positive individuals in mining versus agriculture.

**Figure 5:**
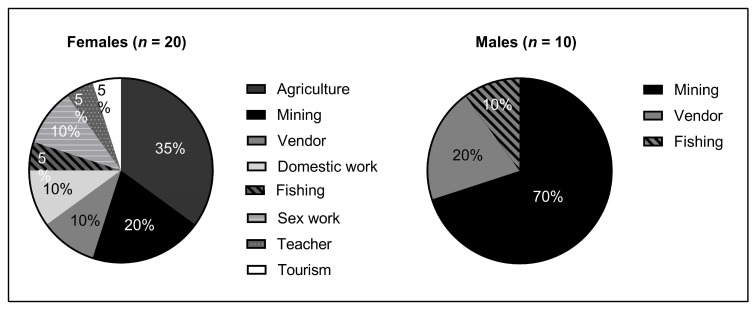
Occupations of HIV-positive individuals divided by gender. Categories of primary employment for individuals who tested positive for HIV.

An additional factor we hypothesized to be associated with HIV status was occupation. Participants in the study practiced 30 unique professions. Patients who tested positive for HIV were found among eight total occupations, of which agriculture and mining encompassed the greatest number of HIV-positive individuals, respectively (Figure 4A). Relatively, HIV prevalence was highest (66.67%) among sex workers, though the sample size of this group was limited (Figure 4B). Although both agriculture and mining are major common occupations in Madagascar, there was a significantly increased relative risk of HIV infection in miners (Figure 4C). Indeed, when segmented by gender, the majority of HIV-positive men practiced mining (7/10), while no male farmers were HIV-positive (Figure 5). In contrast, there was a greater variety of occupations among HIV-positive females.

### 3.3. Presenting Context of HIV-Positive Individuals

Patients enrolled in the study presented for care due to a variety of clinical concerns. The most common presenting chief complaints among HIV-positive patients were another STI (*n* = 8), skin infection (*n* = 7), constitutional symptoms (*n* = 3), and desire for contraception (*n* = 2), respectively. Of these, constitutional symptoms (which included chronic fatigue and weight loss) demonstrated the highest HIV positivity rate of these conditions, with 60% (3/5) of these patients testing positive. Comparatively, 20% (2/10) of those presenting for a contraceptive visit tested positive for HIV (2/10). Among those presenting with suspected skin infections, the majority (4/7) exhibited signs most consistent with a cutaneous fungal rash, while one patient appeared to have shingles, one patient appeared to have scabies, and one patient presented with nonspecific lesions. The remaining HIV-positive individuals were discovered incidentally, endorsing unrelated chief complaints (e.g., toothache, acid reflux) or desiring screening while accompanying another patient or child.

## 4. Discussion

To our knowledge, this study represents the largest general-population HIV prevalence study conducted in Madagascar to date. Using screening as well as confirmatory assays, we observed an HIV prevalence rate, 2.94%, that is substantially higher than the Spectrum model-derived rate of 0.4% thus far utilized by the Malagasy Ministry of Health. This previous figure may have been biased by the scarcity of primary patient test data generated over the past two decades. Our results largely agree with findings reported by the Institut Pasteur in 2002 [6]. Although we observed a higher HIV prevalence compared to 1% seen in the Institut Pasteur study, this increase over two decades could be expected given the low awareness of HIV status among HIV-positive individuals (not currently quantifiable by UNAIDS) and the low rates of HIV-positive individuals who are on active antiretroviral therapy (18%) reported in official estimates [1].

Geography was a key factor we observed to influence HIV prevalence. In comparison to the Institut Pasteur’s study, which focused exclusively on rural individuals, our study included patients from rural villages as well as urban and suburban locales. While the HIV rate we observed in rural patients (2.0%) was closer to the rate observed by the Institut Pasteur in 2002, we noted a six-fold higher rate in urban patients (13.1%) than rural patients, and their inclusion in this study likely accounts for a portion of the elevation in rates observed. This concords with previous studies from other nations across Africa, where higher urban rates of HIV infection have been well-documented [15,16]. We also report a significant association between HIV status and proximity to RN6, the major highway that serves northern Madagascar. Given the evidence of an elevated HIV prevalence in urban centers, we hypothesize that such a thoroughfare might provide a means of HIV dissemination, particularly for individuals who often travel between different towns and cities. The correlation between HIV transmission and routes of travel is a well-established trend that has also been explored in multiple other African nations [17,18]. Yet, to our knowledge, there are few, if any, large-scale efforts to implement general-population HIV screening within urban centers or along main highways. With roughly six Malagasy cities with populations estimated to be 100,000 or greater, there is a large unmet testing need for HIV and other STIs within these localities that could have a watershed impact on nearby suburban and rural communities.

HIV prevalence also varied significantly by occupation, with professional sex work correlating most strongly with HIV-positivity. Interestingly, though, careers such as shopkeeping and tourism also demonstrated a high HIV burden. One theoretical link between these occupations and potential HIV positive status is the need for these individuals to frequently travel to urban centers, whether to restock goods or provide services. In absolute terms, mining had the greatest number of HIV-positive individuals. While this is not surprising given mining is a major industry in Madagascar, this also provides credence to the importance of an individual’s mobility in HIV risk. Miners in Madagascar often move residence based on which locale provides the greatest economic opportunity at a given time, and the correlation between this migratory behavior and HIV prevalence has also been documented in continental Africa [19,20]. Further work is warranted to investigate whether HIV transmission also increases during mining “boom” cycles.

Adding further evidence to these observations, we found that isolation from RN6 and practicing agriculture were negatively correlated with HIV positivity. In fact, no males living in villages away from RN6 and no males who primarily practiced agriculture were found to be HIV-positive in our study, despite large sample sizes. This association was also seen among females, though to a lesser degree. Overall, miners were 2.8 times as likely to test positive for HIV as compared to those with the more stationary occupation of farming. Particularly given the correlation of HIV-positivity with proximity to RN6 in our findings, there is mounting evidence that increased urbanization and roadbuilding throughout rural Madagascar could hasten the dissemination of HIV and other STIs without greater public action, awareness, or preventative strategies. Targeted STI prevention measures should also be considered to specifically target individuals who practice mining and other migratory professions.

Overall, women were 1.5 times as likely as men to test positive for HIV in our study. A contributing explanation is likely the higher physiologic risk of infection faced by women due to increased membrane surface area exposure to infection [21]. Additionally, whereas 70% of HIV-positive males practiced mining, HIV-positive females practiced a much greater variety of occupations than men (Figure 4). A potential factor linking the increased risk of infection faced by women and the greater variety of occupations observed could be the high rates of transactional sex that are especially common and often normalized in rural Madagascar, which often supplement incomes for young females [10,22]. Thus, this additional risk factor could elevate HIV risk regardless of primary occupation. Adding evidence to this, we observed a 66% higher risk of HIV infection in women under the age of 35 as compared to those above this age range and a 3.58 times higher rate of HIV infection in unmarried women as compared to those who were married. These trends were not similar for men, who conversely demonstrated a higher risk of HIV infection with increased age, which may be related to an increased number of sexual encounters with age, potentially due in part to increasing wealth or social status. A related topic we also did not broach in this study is contraception access, which is known to correlate positively with HIV literacy and testing and negatively with HIV prevalence [23]. Recent estimates place the unmet need for contraceptives in Madagascar at roughly 15%, almost double the goal of 9% set by Family Planning 2030, suggesting that additional measures to increase contraceptive access may aid overall HIV control efforts [24,25]. Further studies may benefit from efforts to interview HIV-positive participants about risk factors for HIV acquisition and transmission, including sexual practices, types of relationships, and attitudes towards and usage of contraception. These efforts should also address the stigma of HIV infection among women, another potential barrier to HIV testing and treatment that might also in part contribute to this gender-related HIV prevalence disparity.

In the largely rural population surveyed in this study, 0% of HIV-positive individuals knew their status prior to this investigation, and 0% of HIV-positive individuals were on antiretroviral therapy. Following this investigation, an anthropology team from the University of Antsiranana traveled to Mada Clinics, where they surveyed 96 patients to further understand HIV knowledge. Strikingly, this team found that 60% of those under the age of 18 had never heard of HIV, 78.5% of all respondents did not know the symptoms of HIV, and only 16% of respondents knew that there was a pill available to help treat HIV. Although not specifically quantified, the team observed that knowledge about HIV increased with age, hypothesizing that older individuals may have been exposed to educational HIV campaigns known as “Sensibilisation”, which were common in the early 2000s. Further lending credence to this argument, they reported that several of those surveyed in the older population reported assuming that HIV had been cured as they no longer heard about the illness but had received information about it often in their youth. Beyond the need for expanding testing infrastructure, especially in rural areas, these preliminary data also suggest that more contemporary HIV education initiatives may be necessary to reach the current generation of young Malagasy citizens.

There are a few key limitations to our study. Given the scope of Mada Clinics’ operations and available resources, our study area was limited to northern Madagascar, and although we sampled a geographically varied population across this region, our study did not garner individuals from central or southern Madagascar. Because many cultural and economic distinctions may exist between these regions, the prevalence and rate of HIV transmission may also differ. Furthermore, our testing focused primarily on patients who presented for free, routine primary care. Future efforts might employ screening strategies that are executed directly in the community to further decrease the risk of any sampling bias. With its focus primarily on test data and patient characteristics, our study only captured a proportion of the sociographic data that could be gleaned from HIV-positive individuals. Future studies that combine testing with anthropological survey methodologies might better uncover societal factors to explain the high prevalence of HIV beyond what was gleaned here. Finally, our screening strategy and study scale was limited by the scope of operating within a nonprofit clinic and with limited national resources. HIV testing among the general population should ideally be performed on a regular basis to enable continued surveillance of the status of the potential epidemic. While select individuals within our study also underwent screening for other STIs (e.g., chlamydia, gonorrhea) based on clinical indications, additional efforts are warranted to tie HIV testing to comprehensive evaluation for common and comorbid infections, including syphilis, tuberculosis (TB), and hepatitis B/C. Currently, the only infrastructure for the evaluation of these additional infections within the study area exists at Ministry of Health-sponsored infectious disease clinics in Antsiranana, a journey requiring time and transportation that is frequently out of reach for impoverished patients.

Nevertheless, our findings mark a major advance in the understanding of the extent of HIV across Madagascar. Despite limited HIV data to date, investigators have increasingly begun to question whether a generalized HIV epidemic is on the horizon [5]. Our findings, capturing not only a largely rural population but also individuals from urban centers, strongly suggest that an HIV epidemic is already present within northern Madagascar and may be present across the country as a whole. Indeed, following preprint dissemination of this study, we were contacted by multiple European and Malagasy researchers in western, central, and southern Madagascar who reported emerging data reflecting similar or greater findings to our own. The prospect of rising HIV positivity in Madagascar must also be considered in relation to TB prevalence. Recent estimates from the national tuberculosis surveillance program demonstrate a significant rise in extrapulmonary TB cases, and given the high comorbidity of HIV and TB, this compounds suspicion that underlying HIV prevalence may be growing across the nation as a whole [26]. Of note, TB was considered as a diagnostic possibility for patients who presented with constitutional symptoms in this study, but Mada Clinics currently does not yet possess testing capabilities for mycobacterial infections.

A key contribution of our investigation is the curation of quality general-population HIV testing data, which is important for the success of future HIV prevalence modeling attempts. Ideally, models should not only incorporate this test data but also account for societal trends that drive HIV transmission. A recent example of this was an HIV model designed by a group at the University of Barcelona, who sought to not only incorporate all available testing data, but also to build in Malagasy population-specific variables, including number of sexual partners, rates of transactional sex, and circumcision rate. Employing such a compartmental approach, they concluded that HIV prevalence rates may approach a range of 9 to 24% by 2030 [27]. This alarming conclusion would represent between 3.2 million and 8.54 million HIV infections at Madagascar’s expected 2030 population. If validated, these data suggest that delaying a response to rising HIV rates in Madagascar could spell disastrous consequences and represent one of the largest contemporary public health threats nationwide.

Our findings also directly address multiple gaps in HIV control strategies that were highlighted in a recent Global Fund audit of existing grant-funded programs [9]. While prior efforts have focused exclusively on bridge populations and other groups thought to be “high risk”, our general population screening strategy significantly augments the amount of quality data available to assess general HIV prevalence. By partnering with the Ministry of Health and key stakeholders in the community, we procured sufficient testing supplies, performed confirmatory testing, and referred patients to specialty infectious disease clinics, ensuring that our confirmed HIV-positive patients received correct diagnoses and were connected to adequate levels of care. Furthermore, by focusing on both rural and urban patient populations, our data provide a unique cross-section of HIV positivity in a demographic that likely has previously been missed by efforts located solely in urban locales.

During the process of completing this study, we received numerous requests from other healthcare centers, including national infectious disease clinics, for assistance in providing HIV testing kits. This illustrates the dire need for the expansion of testing infrastructure, as even low-complexity immunoassays, let alone nucleic acid-based viral load tests, are considered scarce resources. This reinforces Global Fund findings that healthcare centers in this region have minimal testing capacity for HIV, further compounding the need for greater investment into testing infrastructure and treatment programs. Based on our findings, we strongly advise the international community to support Madagascar in strengthening HIV testing and treatment programs nationwide. The burden of illness we observed both at Mada Clinics and at hospitals in Antsiranana during this study, as well as patient reports of familial deaths, leads us to conclude that many people are likely dying each day from HIV/AIDS in Madagascar without notice or recognition. The success of programs to combat HIV/AIDS across the rest of the African continent in recent decades clearly demonstrates that such tragedies are avoidable through national and international efforts [2].

## Figures and Tables

**Table 1:** Rapid screening assays for HIV. List of validated diagnostic tests used to initially screen patients for HIV exposure.

Name	Manufacturer	Sample Type (Collection)	Methodology
OraQuick^®^	OraSure Technologies (Bethlehem, PA, USA)	Oral fluid	Immunoassay
HIV-1/2 Antibody Rapid Test	Hightop Biotech (Qingdao, China)	Whole blood (fingerstick)	Immunoassay
Diagnos HIV BI-DOT	J. Mitra & Co. (New Delhi, India)	Whole blood (fingerstick)	Immunoassay
HIV 1.2.O Rapid Test Device	Rapid Labs Ltd. (Essex, UK)	Whole blood (fingerstick)	Immunoassay

**Table 2:** HIV treatment regimens. Antiretroviral medications supplied by the Ministry of Health for the treatment of patients who tested positive.

Name	Medication
Regimen 1	Efavirenz 600 mg
	Lamivudine 300 mg
	Tenofovir disoproxil fumarate 300 mg
Regimen 2	Dolutegravir 50 mg
	Lamivudine 300 mg
	Tenofovir disoproxil fumarate 300 mg

**Table 3:** Patient characteristics and overall HIV prevalence. Demographic information of all patients enrolled in study, including determined HIV status.

Age	
N	1019
Mean (SD)	36.7 (15.2)
Median	34
Range	13–82
**Sex**, n (%)	
Female	577 (56.6%)
Male	442 (43.4%)
**Prevalence**	
HIV-positive	30 (2.9%)
HIV-negative	989 (97.1%)

## Data Availability

Research data available upon request by contacting the lead author.
